# Changes in cerebrospinal fluid synaptic proteins precede and coincide with Alzheimer’s pathology and govern cognitive function

**DOI:** 10.1002/alz.088757

**Published:** 2025-01-09

**Authors:** Hamilton Se‐Hwee Oh, Tony Wyss‐Coray

**Affiliations:** ^1^ Wu Tsai Neurosciences Institute, Stanford University, Stanford, CA USA; ^2^ Department of Neurology and Neurological Sciences, Stanford University School of Medicine, Stanford, CA USA

## Abstract

**Background:**

The buildup of brain amyloid‐beta and tau protein aggregates do not sufficiently explain the heterogeneity in cognitive impairment in Alzheimer’s disease (AD).

**Method:**

To elucidate drivers of cognitive impairment, we measure the levels of 7,000 proteins, in addition to amyloid‐beta‐42 (Ab42) and phospho‐tau‐181 (PTau181), from the cerebrospinal fluid of 2,000 individuals from healthy to severe dementia.

**Result:**

We identify synapse proteins as the strongest correlates of cognitive impairment. We use machine learning methods to derive a cognitive impairment signature, provided as the ratio between YWHAG and NPTX2, which increases with impairment severity (r=0.57) and explains resilience versus susceptibility to AD pathology. We find YWHAG:NPTX2 also increases with age in healthy individuals before PTau181:AB42 and increases 25 years before symptom onset (as early as PTau181:AB42) in autosomal dominant AD.

**Conclusion:**

Our work reveals that synapse alterations precede and coincide with AD pathology accumulation and are the arbiters of cognitive decline. Additionally, we propose the CSF YWHAG:NPTX2 ratio as a biomarker for monitoring synapse health and predicting future cognitive decline in living people.

